# Effects of modular ion-funnel technology onto analysis of breath VOCs by means of real-time mass spectrometry

**DOI:** 10.1007/s00216-020-02846-8

**Published:** 2020-08-13

**Authors:** Giovanni Pugliese, Felix Piel, Phillip Trefz, Philipp Sulzer, Jochen K. Schubert, Wolfram Miekisch

**Affiliations:** 1 Department of Anaesthesia and Intensive Care, Rostock University Medical Center, Schillingallee 35, 18057 Rostock, Germany; 2 IONICON Analytik GmbH, Eduard-Bodem-Gasse 3, 6020 Innsbruck, Austria; 3 Institute for Ion Physics and Applied Physics, University of Innsbruck, Technikerstr. 25, 6020 Innsbruck, Austria; 4 Present Address: Department of Chemistry, University of Oslo, Sem Sælands vei 26, 0371 Oslo, Norway

**Keywords:** PTR-ToF-MS, Ion-funnel, Real-time mass spectrometry, Breath analysis, VOCs

## Abstract

Proton transfer reaction time-of-flight mass spectrometry (PTR-ToF-MS) is a powerful tool for real-time monitoring of trace concentrations of volatile organic compounds (VOCs). The sensitivity of PTR-ToF-MS also depends on the ability to effectively focus and transmit ions from the relatively high-pressure drift tube (DT) to the low-pressure mass analyzer. In the present study, a modular ion-funnel (IF) is placed adjacent to the DT of a PTR-ToF-MS instrument to improve the ion-focusing. IF consists of a series of electrodes with gradually decreasing orifice diameters. Radio frequency (RF) voltage and direct current (DC) electric field are then applied to the electrodes to get the ions focused. We investigated the effect of the RF voltage and DC field on the sensitivity of a pattern of VOCs including hydrocarbons, alcohols, aldehydes, ketones, and aromatic compounds. In a proof-of-concept study, the instrument operating both as normal DT (DC-mode) and at optimal IF conditions (RF-mode) was applied for the breath analysis of 21 healthy human subjects. For the range of investigated VOCs, an improvement of one order of magnitude in sensitivity was observed in RF-mode compared with DC-mode. Limits of detection could be improved by a factor of 2–4 in RF-mode compared with DC-mode. Operating the instrument in RF-mode allowed the detection of more compounds in the exhaled air compared with DC-mode. Incorporation of the IF considerably improved the performance of PTR-ToF-MS allowing the real-time monitoring of a larger number of potential breath biomarkers.

**Electronic supplementary material:**

The online version of this article (10.1007/s00216-020-02846-8) contains supplementary material, which is available to authorized users.

**Figure:**
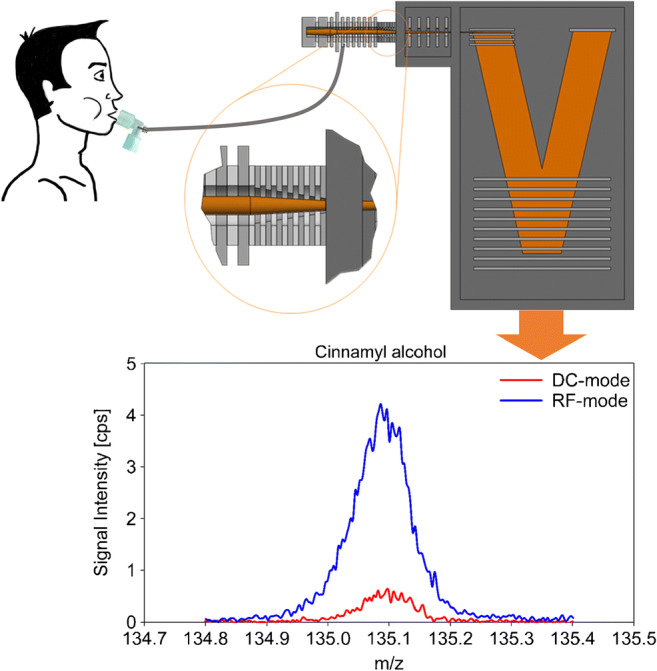
Graphical abstract

## Introduction

Proton transfer reaction mass spectrometry (PTR-MS) is an analytical technique that allows real-time monitoring of volatile organic compounds (VOCs) at low concentrations. It is widely used, e.g. in environmental sciences, food chemistry, homeland security, and breath analysis [1].

Since its introduction in the 1990s [2], PTR-MS has been improved in many ways. Inclusion of time-of-flight (ToF) mass analyzers has substantially overcome the limitations of the first generation of PTR quadrupole-MS (QMS) such as limited mass range and low mass resolution [3–5]. Modifications of the hollow cathode discharge ion source allowed to successfully use different chemical ionization agents such as H_3_O^+^, NO^+^, O_2_^+^, Kr^+^, Xe^+^, and NH_4_^+^, improving versatility and selectivity of the instrument [6, 7].

Sensitivity of PTR-MS is not solely determined by mass analyzers and detectors but it also depends on the ability to effectively focus and transmit ions from the relatively high-pressure drift tube (DT) to the low-pressure mass analyzer. As most of the ions crossing the DT do not exit through the small orifice at the MS interface, a large quantity of ion signal is lost. Ion-funnels (IF) represent a kind of ion guide that enhances sampling of ions through an orifice [8]. In an IF, a radio frequency (RF) voltage and a direct current (DC) electric field are applied to a series of electrodes with decreasing aperture sizes. The electrodes provide strong repulsive potentials at the edge of the electrode, radially focusing the ions. The first demonstration of an IF in PTR-MS was shown by Barber et al. [9]*.* In their instrument, the whole DT was set up as an IF with the first half used as a standard DT reactor running at a lower reduced electric field compared with the traditional DT. The RF electric field was only applied to the second section with decreasing orifice sizes. González-Méndez et al. [10] used the IF to manipulate the ion-molecule reactions and enhance the selectivity of PTR-MS. Brown et al. [11] reported that in this instrument, ion-focusing and proton transfer reaction both occurred in the IF region. This resulted in vastly different sensitivities for different compounds and in unusual fragmentation patterns. Recently, IONICON Analytik implemented a modular IF into proton transfer reaction time-of-flight mass spectrometry (PTR-ToF-MS) instruments. The aim of the present study was to characterize and optimize the IF-PTR-ToF instrument for trace VOC analysis. In a proof-of-concept setup, the instrument was then applied for real-time breath analysis in human subjects. The following questions were addressed in detail:

- How does modification of IF parameters affect primary and VOC product ions?
- Are PTR sensitivity and detection limits in VOC analysis significantly improved by the IF?
- Are benefits of the IF technique suited to support applications such as real-time breath gas analysis in humans?

## Methods

### Ion-funnel PTR-ToF-MS instrument

**Fig. 1:**
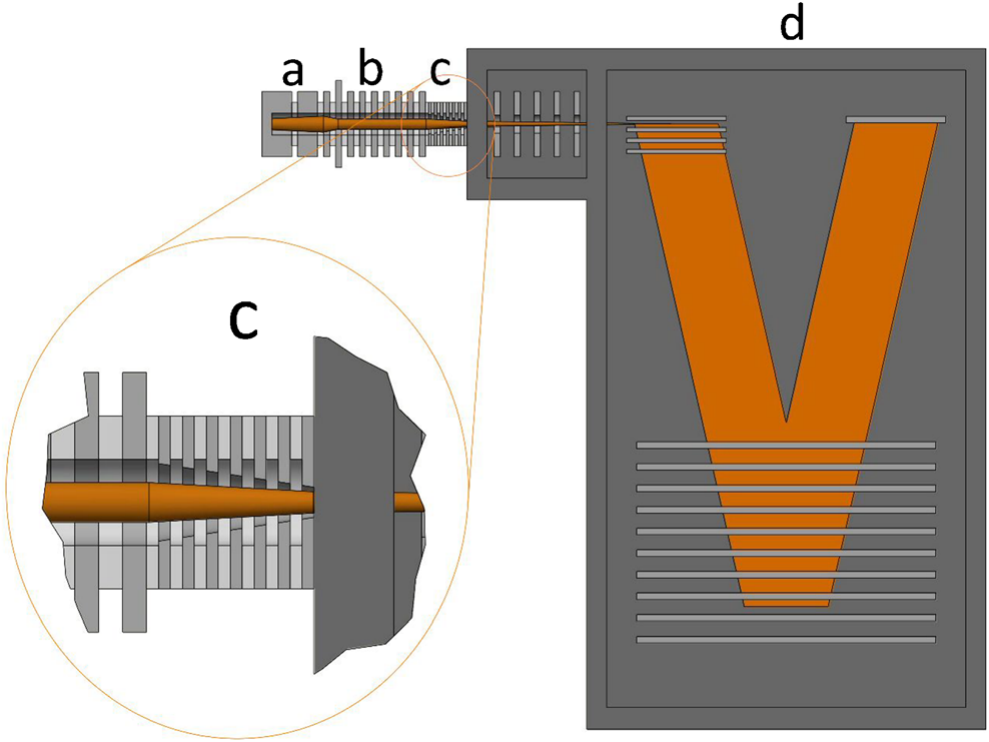
Schematic view of the PTR-ToF 1000 ultra setup: (**a**) hollow cathode ion source, (**b**) drift tube, (**c**) ion-funnel, (**d**) ToF mass analyzer

All investigations were carried out using an online PTR-ToF-MS instrument equipped with a modular IF (PTR-TOF 1000 ultra, IONICON Analytik GmbH, Innsbruck, Austria; first-generation model (2017)). Figure 1 shows a schematic view of the instrument. The general working principle of PTR-ToF-MS has been described in several studies [4, 5]. Concisely, hydronium ions (H_3_O^+^) are produced in a hollow cathode glow discharge ion source from electron ionization of water vapour and are drawn by an electric field into the ion-molecule reaction region (DT). Here, the analyte sample is injected and the proton transfer reaction between the formed H_3_O^+^ and neutral analyte molecules (M) occurs: M + H_3_O^+^ → MH^+^ + H_2_O. Only molecules with proton affinities higher than water (PA (H_2_O) = 691 kJ mol^−1^) are ionized, a criterion that excludes the major constituents of air such as N_2_, O_2_, and CO_2_ but includes many trace gases such as most VOCs. The DT of the PTR-TOF 1000 ultra consists of a 7 cm long tube made of electrically isolated stainless steel rings. The rings are connected with resistors, and a drift voltage (*U*_drift_) can be applied over the entire set of rings to induce an electric field (*E*_drift_) in the DT. The modular IF is 2.2 cm long, it is placed adjacent to the DT and consists of 12 electrodes (6 with RF+ and 6 with RF−) with gradually decreasing orifice diameters, from 1 to 0.2 cm, placed at 0.1 cm distance of each other. In order to avoid the trapping of ions in axial potential wells, particularly those with low *m*/*z*, the IF electrode geometries used in this work fulfilled the following conditions:

$$2\uppi \frac{\rho }{\delta}\exp \left(\frac{-2\rho }{\delta}\right)\ll 1$$

where *ρ* is the electrode orifice radius and *δ* = *d*/π where *d* is the electrode spacing [12]. A radio frequency (RF) voltage and a direct current (DC) electric field are then applied to the electrodes. The DC electric field drives the ions axially through the IF toward the exit aperture. An additional alternating current (AC) is superimposed on the electrodes, with the RF on neighbouring electrodes being phase-shifted by 180°. In this way, the RF field creates a strongly repulsive potential near the surface of each electrode. In combination with the progressively decreasing aperture size, this serves to focus the ions radially. Table S1 (see Electronic Supplementary Material, ESM) summarizes details and operating conditions of the modular IF. The protonated VOCs then enter the pulse extraction region of the orthogonal acceleration reflectron ToF analyzer via a transfer lens system. The DT is interfaced to the transfer lens region via a pinhole of ~ 0.1 cm I.D. with the cone toward the transfer lens. The operating pressure in the DT (buffer gas number density, *N*) and the *E*_drift_ strength are important parameters, more commonly combined and expressed in terms of the reduced electric field (*E*/*N*). *E*_drift_ accelerates the ions but at the same time collisions with the buffer gas tend to slow them down. The *E*/*N* affects the reagent ion distribution. Increasing the *E*/*N* ratio results in more energetic collisions, which reduces the proportion of the water cluster ions (H_3_O^+^(H_2_O)_*n*_) in the DT but at the same time can increase the fragmentation of analytes [13]. Typical *E*/*N* values are in the range 90–150 Td, where 1 Td = 10^−17^ V cm^2^.

Within the standard PTR DT, *E*_drift_ variation is about 15% due to the ratio of the inner diameter and axial distance between the drift rings [14]. The additional RF voltage considerably increases this variation and *E*/*N* cannot be properly calculated any more. However, it is possible to define certain voltage settings in RF-mode which enable branching ratios of distinct analytes to be obtained that are comparable with the ones obtained with a classic PTR-ToF-MS instrument operated at a certain actual *E*/*N* (“TRU-*E*/*N*” method). Nevertheless, in the present paper, the *E*/*N* definition is omitted.

### Ion-funnel characterization

#### Gas standard generation

The IF characterization was performed using a multicomponent gas VOC mix (IONICON Analytik GmbH, Innsbruck, Austria) including methanol, acetonitrile, acetaldehyde, ethanol, 2-propenal, acetone, isoprene, 2-butenal, 2-butanone, benzene, toluene, *o*-xylene, chlorobenzene, *α*-pinene, and 1,2-dichlorobenzene at concentrations of ~ 1ppmV. The VOC mix was subjected to a 100-fold dynamic dilution in pure nitrogen (purity 5.0, Linde, Vienna, Austria) by means of a liquid calibration unit (LCU, IONICON Analytik, Innsbruck, Austria) to generate a standard mixture with approximately 10 ppbV of each component. The working principle of the LCU involves the introduction of a liquid standard solution into a carrier gas stream, by forcing it through a nebuliser (X175, Burgener Research Inc., UK) and spraying the solution into an evaporation chamber at a defined temperature. This results in a rapid evaporation. The generated gaseous standard mixture can then be measured or collected directly at the output of the evaporation chamber. Two liquid ports (1–50 μl min^−1^), one carrier gas port (1–1000 ml min^−1^), and two additional gas ports (1–100 ml min^−1^), controlled by mass flow controllers (Bronkhorst High-Tech B.V., Ruurlo, Netherlands), enable the generation of complex standard mixtures. VOC standards can be prepared from either liquid solutions or gases, or even from both at the same time. In addition, defined amounts of humidity can be added by adding pure water via one of the two liquid ports [15].

In this study, the LCU flow was kept constant at 1000 ml min^−1^ for all experiments, the LCU temperature was 75 °C and the humidity was adjusted by adding pure water (HPLC grade).

#### Experimental design

The standard mixture was introduced into the DT via a 1.5 m long polyether ether ketone (PEEK) transfer line (ID: 0.75 mm, Restek, Bellafonte, PA) that was directly connected to the outlet of the LCU. The transfer line temperature was 75 °C and the sampling flow was 100 ml min^−1^. The signal intensity was recorded for each *m*/*z* while the settings of the IF region were varied. Operating the instrument in RF-mode (RF on), the DC electric field applied to the IF was varied in the range of 4.5–27 V/cm while the RF voltage was varied in the range of 40–200 V peak-to-peak (V_p-p_) at 4.5 MHz. These testing ranges were decided upon after preliminary measurements showed that these settings approximate the best operating conditions. The entire experimental design was repeated at two different *E*_drift_ strength, 66 V/cm and 48 V/cm, and using both dry and humid samples (absolute humidity 47 g m^−3^) (ESM Table S2). These two sampling conditions will be referred in the text as “dry” and “humid” conditions, respectively.

When the instrument was operated only with the DC field applied to the IF region (DC-mode), the RF voltage was set to zero and the DC field was set at the same *E*_drift_ value in order to have a homogeneous electric field along the DT/IF regions.

For the whole experimental design, the DT/IF pressure was 2.3 mbar, the DT temperature was 75 °C, and the integration time was 1 s.

Three replicates were measured for each experimental setup, then the results were averaged and background signals were subtracted.

### Human breath samples

All experiments were performed in accordance with the guidelines laid down in the Declaration of Helsinki and approved by the ethics committee at the University Medical Center Rostock. Informed consent was obtained from 21 healthy human subjects (aged between 20 and 45 years). Demographic parameters such as height, body weight, age, sex, and smoking habits were recorded for each volunteer (ESM Table S3). Volunteers were asked to breathe spontaneously and continuously over 3 min through a sterile mouth piece directly connected to the PTR transfer line in side stream mode by means of a T-piece. During the first minute of measurement, the PTR-ToF-MS instrument was operated in RF mode: *E*_drift_ was 66 V/cm, RF voltage was 120 V_p-p_, and DC field was 13.5 V/cm. During the second minute of measurement, the operating mode of the instrument was switched from RF-mode to DC-mode. During the third minute of measurement, the instrument was operated in DC-mode: RF voltage was 0 V_p-p_ and both *E*_drift_ and DC field were 66 V/cm.

For breath measurements, the PTR transfer line temperature was 75 °C, DT temperature was 75 °C, and DT pressure was 2.3 mbar. For breath measurements, the integration time was 200 ms.

### Data processing

The ion yields of all *m*/*z* were measured in counts per second (cps) and compounds were identified by means of their protonated *m*/*z* and isotopic patterns. The normalization of the measured ion intensities to the H_3_O^+^ counts in combination with the water-cluster ion counts is standard practice in PTR-ToF-MS data treatment [16]. However, in the present paper, the normalization to reagent ions was omitted in order to reflect the actual sensitivity of the instrument, which would be masked by normalization.

Both breath and standard files were processed using the software PTR-MS viewer v. 3.2.8 (IONICON Analytik GmbH, Innsbruck, Austria).

For breath measurements, expiratory and inspiratory phases were recognized by means of a custom-made data processing algorithm called “breath tracker” (MATLAB version 7.12.0.635, R2011a). The function of the algorithm has been described previously [17]. Briefly, an endogenous compound that has a sufficiently abundant signal intensity in expiration is used as a tracker to differentiate between alveolar and inspired phases. Acetone, isoprene, or carbon dioxide is usually used for this purpose. Expiratory and inspiratory phases determined by means of the algorithm were then applied to all *m*/*z* of interest.

## Results

### Ion-funnel characterization

#### H_3_O^+^·(H_2_O)_*n*_ (*n* = 0, 1, and 2) reagent ions

**Fig. 2:**
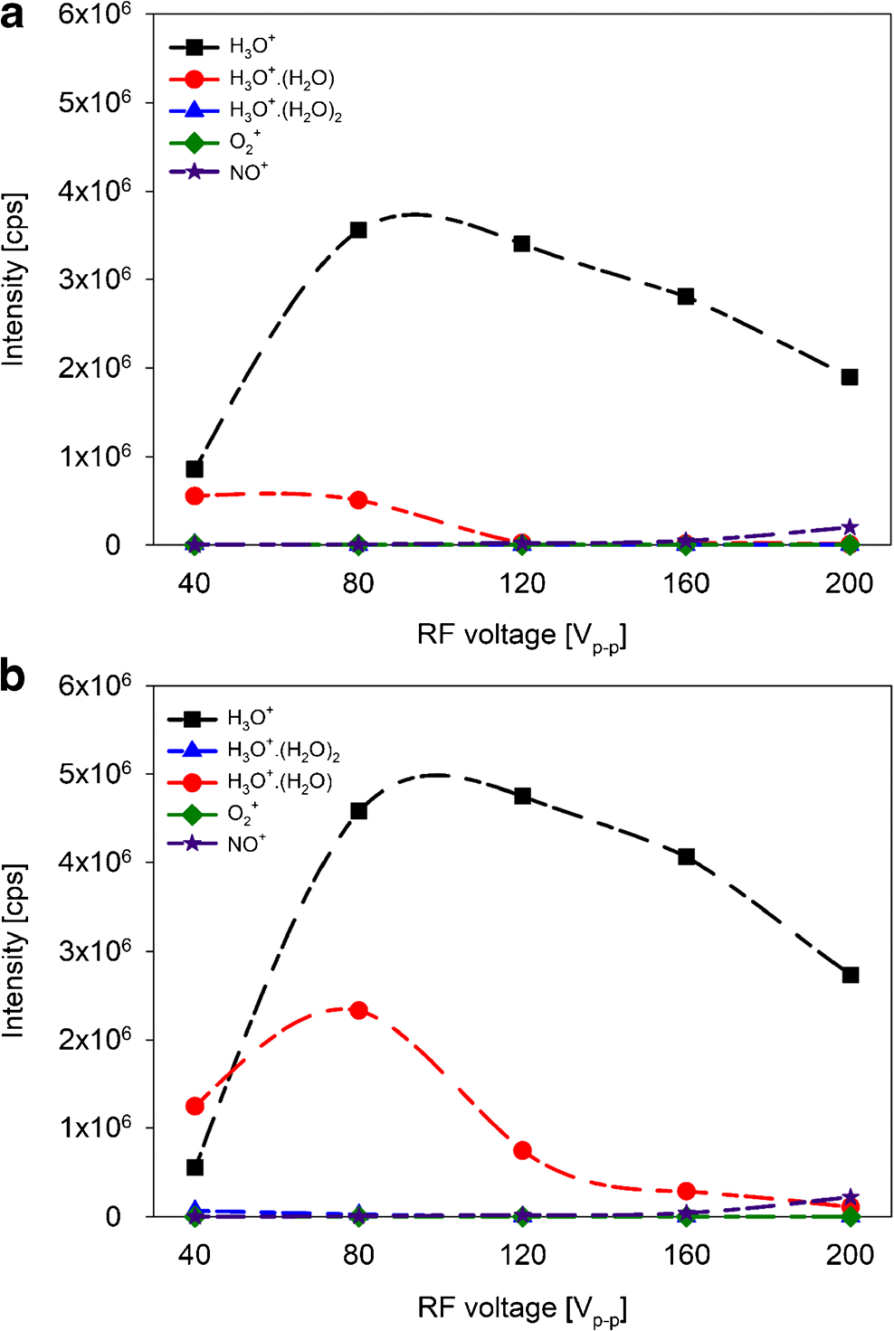
Ion intensities in counts per second (cps) of the water reagent ions (H_3_O^+^·(H_2_O)_*n*_, *n* = 0, 1, and 2) and parasitic ions O_2_^+^ and NO^+^ present in the DT under dry (**a**) and humid (**b**) conditions as a function of RF voltage. DC was 13.5 V/cm and *E*_drift_ was 66 V/cm

Figure 2 (a, b) shows the variation of H_3_O^+^, protonated water clusters, and O_2_^+^ and NO^+^ measured intensities as function of RF voltage. The signal intensities of H_3_O^+^, H_2_O·H_3_O^+^, and O_2_^+^ are too high to be measured directly because of detector saturation. Therefore, the signal intensities at *m*/*z* = 21 corresponding to H_3_^18^O^+^, at *m*/*z* = 39 corresponding to H_2_O·H_3_^18^O^+^ and at *m*/*z* = 34 corresponding to ^18^O^16^O^+^ were recorded and corrected by the natural isotope abundances.

H_3_O^+^ intensity showed its maximum value at RF = 80 V_p-p_ under dry conditions (Fig. 2 (a)) and at RF = 120 V under humid conditions (Fig. 2 (b)). As the RF voltage decreases, the H_3_O^+^ signal intensity decreases. At the same time, an increase of the water cluster intensities was observed with decreasing RF voltage. H_3_O^+^·H_2_O showed its maximum value at RF = 40 V_p-p_ under dry conditions and at RF = 80 V_p-p_ under humid conditions. Nevertheless, at RF = 40 V_p-p_ under humid conditions, H_3_O^+^·H_2_O showed a higher intensity than H_3_O^+^.

The protonated water trimer was only observed under humid conditions and at RF = 40 V_p-p_. Intensities of parasitic ions O_2_^+^ and NO^+^ only increased only at high RF voltages (> 160 V_p-p_) under both dry and humid conditions. H_3_O^+^·(H_2_O)_2_ and O_2_^+^ intensities were up to six orders of magnitude smaller compared with that of the protonated water dimer.

**Fig. 3:**
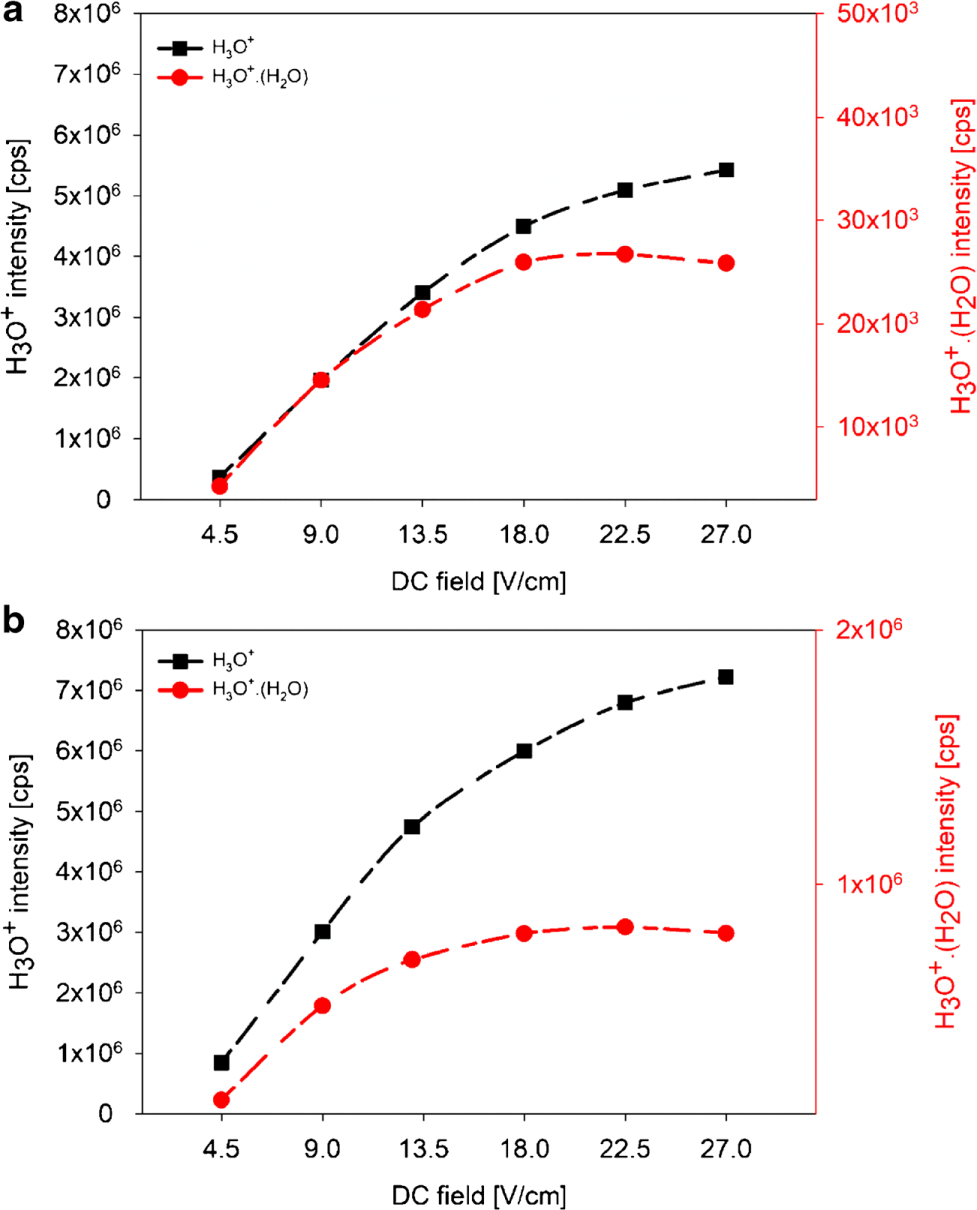
Ion intensities in counts per second (cps) of H_3_O^+^ and H_3_O^+^·(H_2_O) present in the DT under dry (**a**) and humid (**b**) conditions as a function of the DC field (V/cm). RF voltage was 120 V_p-p_ and *E*_drift_ was 66 V/cm

Figure 3 (a, b) shows the variation of the H_3_O^+^ and H_3_O^+^·H_2_O intensities as function of DC field. Due to the large difference between the intensities of the two reagent ions, H_3_O^+^·H_2_O intensity is displayed on a second *Y*-axis. The H_3_O^+^ intensity increased with increasing DC field with its maximum value at DC = 27 V/cm under both dry (Fig. 3 (a)) and humid (Fig. 3 (b)) conditions. In contrast, H_3_O^+^·H_2_O showed its maximum value at DC = 22.5 V/cm under both dry and humid conditions.

ESM Fig. S1 (a, b) shows the variation of H_3_O^+^, protonated water clusters, and O_2_^+^ and NO^+^ measured intensities as function of RF voltage at *E*_drift_ = 48 V/cm. ESM Fig. S2 (a, b) shows the variation of the H_3_O^+^ and H_3_O^+^·H_2_O intensities as function of DC voltage at *E*_drift_ = 48 V/cm. H_3_O^+^ intensity showed similar trends of those showed at *E*_drift_ = 66 V/cm. In contrast, substantial differences were found for the protonated water clusters. Under dry conditions (ESM Fig. S1 (a)) at RF = 40 V_p-p_ and under humid conditions (ESM Fig. S1 (b)) at RF = 80 V_p-p_, H_3_O^+^·H_2_O became the most abundant reagent ion in the DT. Under humid conditions at RF = 40 V_p-p_, H_3_O^+^·(H_2_O)_2_ showed a higher intensity than H_3_O^+^.

In DC-mode at *E*_drift_ = 48 V/cm under humid conditions, H_3_O^+^·H_2_O represent about 65% of the total water reagent ions. In contrast, under dry conditions, they represent about the 15% of the total water reagent ions. In DC-mode at *E*_drift_ = 66 V/cm, protonated water clusters are present in low concentrations under both dry and humid conditions. Under humid conditions, H_3_O^+^·H_2_O represent about 8% of the total water reagent ions; under dry conditions, they represent about 1% of the total water reagent ions.

#### Effect of RF voltage and DC field on VOC signal intensities

**Fig. 4:**
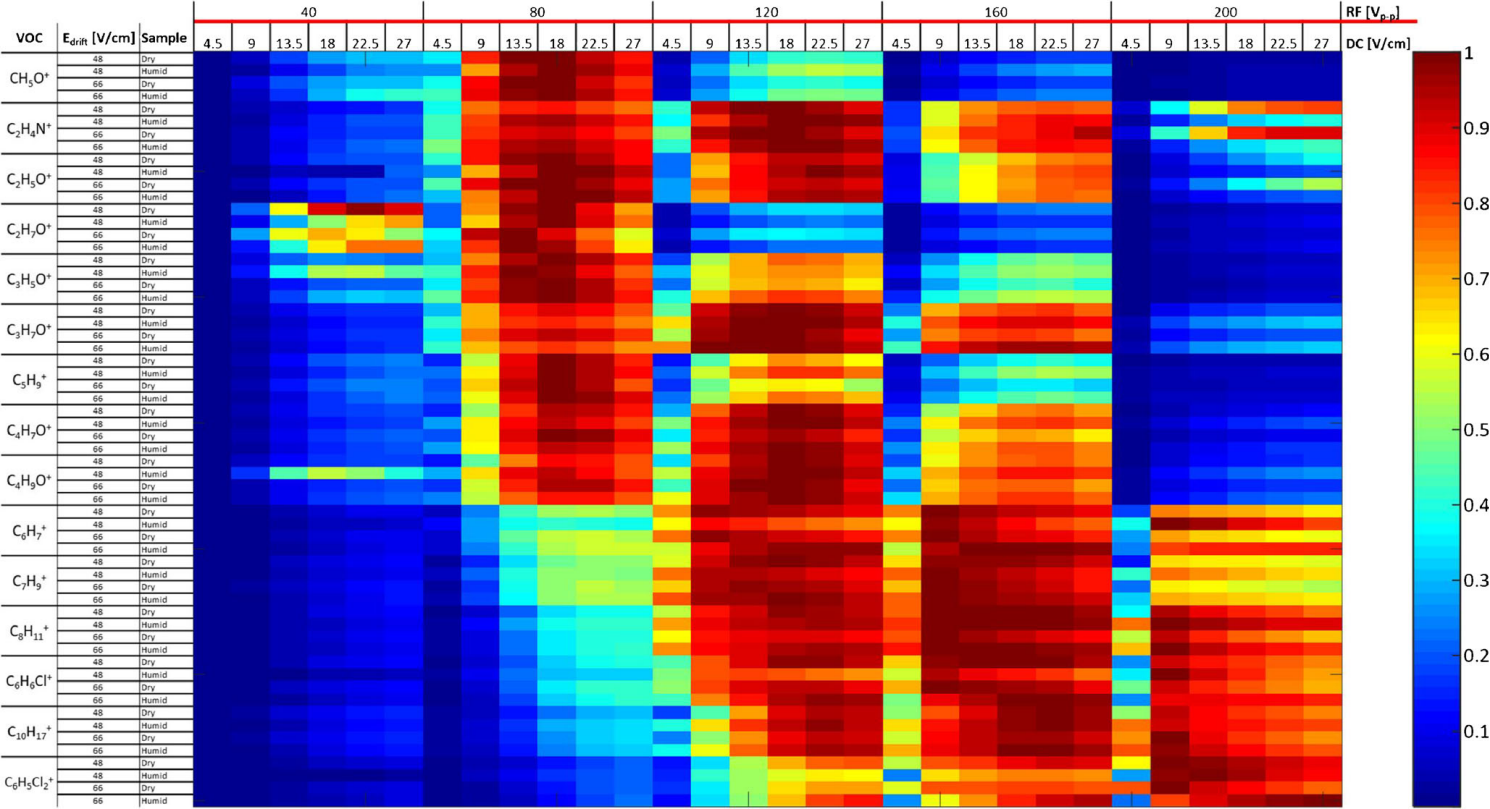
Effect of RF voltage (40–200 V_p-p_) and DC field (4.5–27 V/cm) on VOC intensities. The whole experiment was conducted at *E*_drift_ of 48 V/cm and 66 V/cm, with dry and humid samples (humidity of 47 g m^−3^). Data were normalized to maximum values to emphasize relative changes

Figure 4 shows effects of RF voltage (40–200 V_p-p_) and DC field (4.5–27 V/cm) onto intensities of all investigated VOCs.

**Fig. 5:**
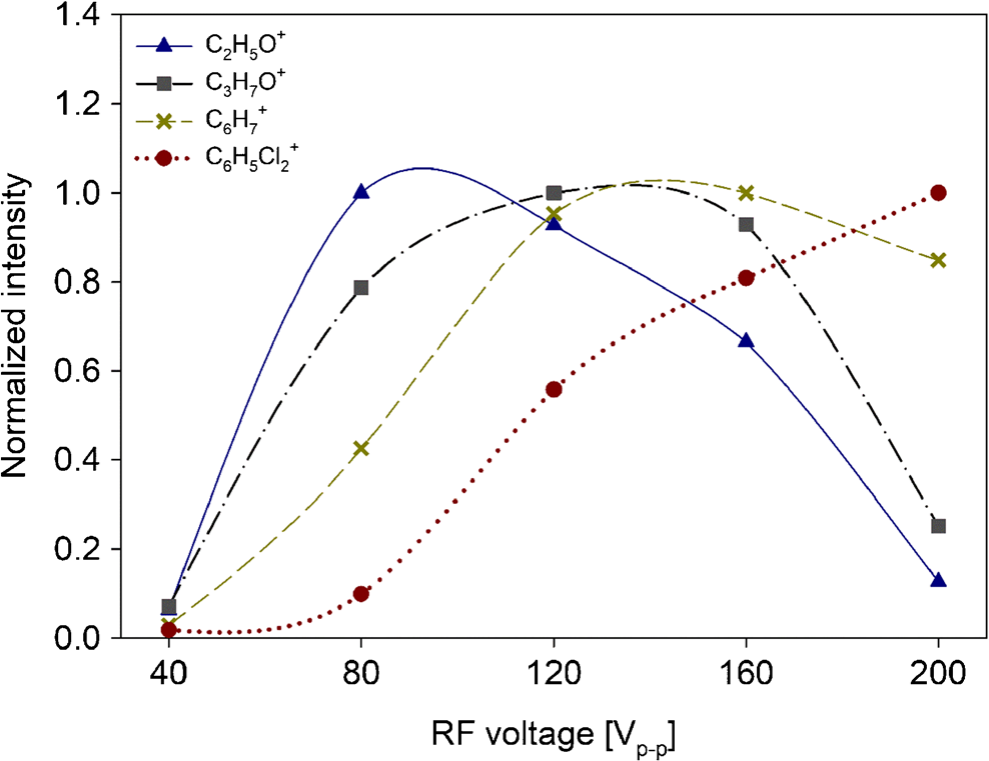
Intensities of acetaldehyde (blue triangle), acetone (grey square), benzene (yellow cross), and dichlorobenzene (red round) as function of RF voltage. DC field was 13.5 V/cm and *E*_drift_ was 66 V/cm. Data were normalized onto respective maximum values to emphasize the relative changes

Figure 5 shows signal intensities of acetaldehyde, acetone, benzene, and dichlorobenzene as a function of RF voltage.

Intensities of acetaldehyde, methanol, ethanol, 2-propenal, and isoprene showed their maximum values at RF = 80 V_p-p_. In contrast, intensities of acetone, acetonitrile, 2-butenal, and butanone showed their maximum values at RF = 120 V_p-p_. Aromatic compounds, such as benzene, toluene, *o*-xylene, chlorobenzene, and α-pinene, showed their maximum intensities at RF = 160 V_p-p_. Intensity of dichlorobenzene showed its maximum at RF = 200 V_p-p_.

**Fig. 6:**
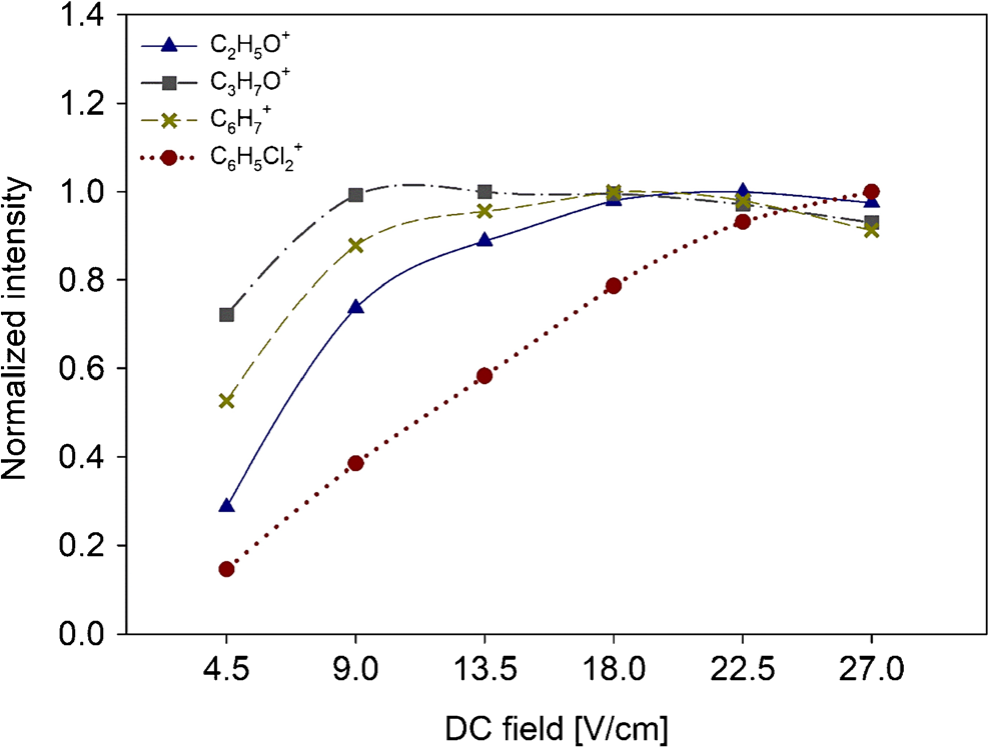
Intensities of acetaldehyde (blue triangle), acetone (grey square), benzene (yellow cross), and dichlorobenzene (red round) as function of DC voltage. RF voltage was 120 V_p-p_ and *E*_drift_ was 66 V/cm. Data were normalized onto respective maximum values to emphasize the relative changes

Figure 6 shows the measured intensities of the acetaldehyde, acetone, benzene, and dichlorobenzene as function of DC field. Intensities of most of the investigated compounds showed a substantial increase when the DC field was increased from 4.5 to 13.5 V/cm; when the DC voltage was further increased up to 27 V/cm, they showed variations < 10%. This was with the exception of dichlorobenzene which showed steadily increasing intensity with increasing DC voltage, with its maximum at DC = 27 V/cm.

Table 1 compares sensitivities and limits of detection (LODs) calculated for DC-mode and RF-mode for all the investigated VOCs. Sensitivity is expressed as ion count rate per second per part-per-billion volume mixing ratio of supplied analyte (cps/ppbV). LODs were calculated for 1 s of integration time using the “3*σ* method” with *σ* being the standard deviation of the background noise level [18]. The DC-mode data were collected at *E*_drift_ = 66 V/cm, RF = 0 V_p-p_, and DC = 66 V/cm under humid conditions. The RF-mode data were collected at *E*_drift_ = 66 V/cm, RF = 120 V_p-p_, and DC = 13.5 V/cm under humid conditions. At these conditions, switching from DC-mode to RF-mode led to an improvement in sensitivity of about 1 order of magnitude for most of the investigated compounds with the exception of methanol, ethanol, and dichlorobenzene. In contrast, only an improvement by a factor of 2–4 was observed for the LODs in RF-mode compared with DC-mode.

**Table 1:** Comparison of sensitivities and LODs for DC-mode and RF-mode of all investigated VOCs. LODs were calculated for 1 s of integration time. The DC-mode data were collected at *E*_drift_ = 66 V/cm, RF = 0 V_p-p_, and DC = 66 V/cm under humid conditions. The RF-mode data were collected at *E*_drift_ = 66 V/cm, RF = 120 V_p-p_, and DC = 13.5 V/cm under humid conditions

	Sensitivity (cps ppbV^−1^)			LOD (ppbV)		
Compound (*m*/*z*)	DC-mode	RF-mode	RF-mode/DC-mode	DC-mode	RF-mode	RF-mode/DC-mode
Methanol (33)	27.5	116.5	4.2	0.735	0.432	1.7
Acetonitrile (42)	46.7	418.2	8.6	0.337	0.11	3.1
Acetaldehyde (45)	56.5	458.1	8.1	0.66	0.245	2.7
Ethanol (47)	3.4	18.2	5.3	12.319	8.31	1.5
2-Propenal (57)	43	406.3	9.4	0.301	0.146	2.1
Acetone (59)	103.6	1008.3	9.7	0.26	0.09	2.9
Isoprene (69)	10	120.8	12.1	1.226	0.463	2.6
2-Butenal (71)	65.8	701	10.7	0.163	0.069	2.4
2-Butanone (73)	66.1	750.9	11.4	0.325	0.104	3.1
Benzene (79)	39.2	423.6	10.8	0.22	0.056	3.9
Toluene (93)	50.2	620.3	12.4	0.144	0.06	2.4
*o*-Xylene (107)	65.4	747.4	11.4	0.115	0.03	3.8
Chlorobenzene (113)	38.2	413.7	10.8	0.155	0.061	2.5
*α*-Pinene (137)	25.7	252.1	9.8	0.265	0.087	3
1,2-Dichlorobenzene (147)	43.9	264.7	6.0	0.087	0.044	2

### Application in human breath samples

In a proof-of-concept study, the instrument operating both in DC-mode and RF-mode was applied for breath analysis of 21 human healthy subjects.

Table 2 contains the list of VOCs that could be detected in exhaled breath. Compounds that could only be detected in RF-mode are labelled using bold italic text. Concentrations of these compounds were below the LODs in DC-mode and above the LODs in RF-mode.

**Table 2:** List of VOCs that could be detected from exhaled breath in real-time. Compounds that could only be detected in RF-mode compared with DC-mode are labelled using bold italic text. The DC-mode data were collected at *E*_drift_ = 66 V/cm, RF = 0 V_p-p_, and DC = 66 V/cm. The RF-mode data were collected at *E*_drift_ = 66 V/cm, RF = 120 V_p-p_, and DC = 13.5 V/cm

Peak number	Measured mass (*m*/*z*)	Exact mass (*m*/*z*)	Mass accuracy (ppm)	Empirical formula	Concentration range (ppbV)	LOD DC-mode (ppbV)	LOD RF-mode (ppbV)	LOQ DC-mode (ppbV)	LOQ RF-mode (ppbV)	Potential compound
1	33.031	33.033	− 60.55	CH_4_O^+^	37.401–364.033	0.735	0.432	2.426	1.426	Methanol
2	42.031	42.034	− 71.37	C_2_H_4_N^+^	2.443–76.18	0.337	0.11	1.112	0.363	Acetonitrile
3	47.046	47.049	− 63.76	C_2_H_6_O^+^	12.332–74.094	12.319	8.31	40.653	27.423	Ethanol
4	59.053	59.049	67.74	C_3_H_7_O^+^	133.39–1043.864	0.26	0.09	0.858	0.297	Acetone
5	61.031	61.028	49.16	C_2_H_5_O_2_^+^	4.328–40.298	0.865	0.763	2.855	2.518	Acetic acid
6	63.028	63.026	31.73	C_2_H_7_S^+^	1.435–10.894	0.203	0.12	0.67	0.396	Dimethylsulfide
7	69.072	69.070	28.96	C_5_H_9_^+^	31.166–193.766	1.226	0.463	4.046	1.528	Isoprene
8	71.052	71.049	42.22	C_4_H_7_O^+^	0.25–6.957	0.163	0.069	0.538	0.228	2-Butenal
9	73.069	73.065	54.75	C_4_H_9_O^+^	1.26–3.769	0.325	0.104	1.073	0.343	Butanone
10	79.052	79.054	− 25.30	C_6_H_7_^+^	0.309–8.362	0.22	0.056	0.726	0.185	Benzene
11	87.073	87.080	− 80.39	C_5_H_11_O^+^	0.62–1.643	0.349	0.272	1.152	0.898	Pentanal
12	89.052	89.060	− 89.83	C_4_H_9_O_2_^+^	0.469–5.146	0.203	0.126	0.67	0.416	Ethylacetate
13	93.073	93.070	32.23	C_7_H_9_^+^	0.413–7.61	0.144	0.06	0.475	0.198	Toluene
14	137.129	137.132	− 21.88	C_10_H_17_^+^	0.911–12.413	0.265	0.087	0.875	0.287	Limonene
***15***	***49.008***	***49.010***	***− 40.81***	***CH***_***5***_***S***^***+***^	***0.194–2.452***	***0.354***	***0.073***	***1.168***	***0.241***	***Methyl mercaptan***
***16***	***68.053***	***68.049***	***58.78***	***C***_***4***_***H***_***6***_***N***^***+***^	***0.067–0.558***	***0.141***	***0.032***	***0.465***	***0.106***	***Pyrrole***
***17***	***80.047***	***80.049***	***− 24.98***	***C***_***5***_***H***_***6***_***N***^***+***^	***0.068–0.713***	***0.215***	***0.039***	***0.71***	***0.129***	***Pyridine***
***18***	***85.063***	***85.065***	***− 23.51***	***C***_***5***_***H***_***9***_***O***^***+***^	***0.331–1.218***	***0.323***	***0.266***	***1.066***	***0.878***	***3-Penten-2-one***
***19***	***91.055***	***91.057***	***− 21.96***	***C***_***4***_***H***_***11***_***S***^***+***^	***0.168–1.091***	***0.263***	***0.092***	***0.868***	***0.304***	***Methyl propyl sulfide***
***20***	***97.061***	***97.065***	***− 41.21***	***C***_***6***_***H***_***9***_***O***^***+***^	***0.248–3.97***	***0.334***	***0.13***	***1.102***	***0.429***	***2,5-Dimethylfuran***
***21***	***101.055***	***101.060***	***− 49.48***	***C***_***5***_***H***_***8***_***O***_***2***_^***+***^	***0.218–0.737***	***0.261***	***0.195***	***0.861***	***0.644***	***Coffee furanone***
***22***	***105.056***	***105.054***	***19.04***	***C***_***4***_***H***_***9***_***O***_***3***_^***+***^	***0.142–0.785***	***0.242***	***0.12***	***0.799***	***0.396***	***β-Hydroxybutyric acid***
***23***	***106.069***	***106.065***	***37.71***	***C***_***7***_***H***_***8***_***N***^***+***^	***0.017–0.094***	***0.088***	***0.05***	***0.29***	***0.165***	***Vinylpyridine***
***24***	***108.074***	***108.081***	***− 64.77***	***C***_***7***_***H***_***10***_***N***^***+***^	***0.063–0.242***	***0.202***	***0.077***	***0.667***	***0.254***	***o-Toluidine***
***25***	***118.065***	***118.058***	*** 59.29***	***C***_***8***_***H***_***8***_***N***^***+***^	***0.051–0.569***	***0.084***	***0.014***	***0.277***	***0.046***	***Indole***
***26***	***125.088***	***125.096***	***− 63.95***	***C***_***8***_***H***_***13***_***O***^***+***^	***0.042–0.343***	***0.168***	***0.089***	***0.554***	***0.294***	***Acetylcyclohexene***
***27***	***129.077***	***129.070***	***54.23***	***C***_***10***_***H***_***9***_^***+***^	***0.07–0.153***	***0.161***	***0.059***	***0.531***	***0.195***	***Naphthalene***
***28***	***133.058***	***133.065***	***− 52.61***	***C***_***9***_***H***_***9***_***O***^***+***^	***0.042–0.142***	***0.12***	***0.04***	***0.396***	***0.132***	***Cinnamaldehyde***
***29***	***135.088***	***135.080***	***59.22***	***C***_***9***_***H***_***11***_***O***^***+***^	***0.074–0.317***	***0.167***	***0.075***	***0.551***	***0.248***	***Cinnamyl alcohol***
***30***	***149.055***	***149.060***	***− 33.54***	***C***_***9***_***H***_***9***_***O***_***2***_^***+***^	***0.095–0.561***	***0.223***	***0.126***	***0.736***	***0.416***	***Cinnamic acid***
***31***	***151.110***	***151.112***	***− 13.24***	***C***_***10***_***H***_***15***_***O***^***+***^	***0.054–0.233***	***0.118***	***0.049***	***0.389***	***0.162***	***Carvone***

Concentrations and LODs and LOQs were calculated applying the kinetic theory [19, 20].

## Discussion

Incorporation of a modular IF adjacent to the DT led to a substantial improvement in sensitivity and LODs of the PTR-ToF-MS instrument. Improved sensitivities allowed the detection of a broader range of VOCs from human breath samples in real-time.

Intensities determined for water reagent ions (H_3_O^+^·(H_2_O)_*n*_, *n* = 0, 1, and 2) and for protonated VOCs showed a considerable dependence on RF voltage and DC field applied to the IF region. Highest intensities for H_3_O^+^ were observed in the RF range 80–120 V_p-p_ and at DC = 27 V/cm. At lower and higher RF voltages, the focusing effect of the funnel was lost and ion transfer was less efficient. High RF voltages increase the kinetic energy of molecules. As binding forces in the water clusters are weak when compared with normal chemical bonding, this will lead to collisional decomposition of water clusters long before fragmentation of chemical compounds occurs. Higher H_3_O^+^ intensities in humid samples were most probably due to back diffusion of sample gas from the DT into the ion source generating additional H_3_O^+^ [15, 21, 22]. O_2_^+^ and NO^+^ were present in low intensities and were observed in RF-mode only at high RF voltage (> 160 V_p-p_) as a result of improved ion transmission [13, 23].

VOCs showed maximum intensities at substance-specific DC field and RF voltage. In agreement with IF theory, cut-off values occurred at low (< 50 Vp-p) RF voltages for all VOCs and at high (> 160 Vp-p) RF voltages for low-mass compounds (*m*/*z* < 90). At low RF voltages, the focusing effect of IF is lost for low and high masses. In a substance-specific way, higher masses show maximum transmission at high (> 120 Vp-p) RF voltages due to the dependency of effective potential onto *m*/*z*. Decreasing transmission of low masses at high RF voltages is attributed to diffusional loss of molecules due to the relatively high kinetic energy of low-mass molecules under these conditions [12, 24]. In addition, fragmentation may contribute to this effect, as we observed a 10% increase in acetaldehyde fragmentation with increasing RF voltage. Up to 50% fragmentation was reported by Barber et al. under similar conditions.

In contrast to oxygen-containing aliphatic substances, aromatic compounds showed increasing ion yields of the protonated monomers even at high RF voltages where non-aromatic substances already exhibited decreasing intensities. Enhanced ability of aromatic systems to stabilize ionic states may explain efficient generation of molecule ions without losses through fragmentation even at high RF voltages. This hypothesis is further confirmed by dichlorobenzene showing an almost linear increase with increasing RF voltages, most probably due to the additional charge-stabilizing effects of the chlorine atoms.

In contrast to previous setups, with the IF used in this study, sensitivity increases were rather uniform, i.e. approximately one order of magnitude for all investigated compounds. This is a strong indicator that the IF does not have major effects onto the ion chemistry within the DT itself. Therefore, the advantages of PTR-MS, e.g. quantification without calibration, are preserved. Although Brown and Barber et al. reported relative increases in sensitivity of 1–2 orders of magnitude for single compounds (acetaldehyde 45×, acetone 200×), absolute sensitivities for a broad range of compounds reported in our study were in general higher, e.g. 10 times higher for methanol and 2 times higher for acetaldehyde. Higher improvements in relative sensitivity as well as higher fragmentation rates reported by Barber and Brown et al. can thus be attributed to different geometries of IF and DT in their instruments.

Characterization and optimization of DT conditions, RF voltage, and DC field and effects of humidity are of general importance for any IF-PTR-ToF instrument and can therefore be beneficial for the whole community [20]. In addition, the modular IF described in this study can be implemented into several PTR-ToF-MS instruments.

The impact of the IF onto quantification can be seen when LODs and LOQs are looked upon. As the applied IF will improve transmission of the ions, in parallel to the desired effects, increased ion yields will also induce growing background noise. Thus, the “raw” gain in ion counts will not directly translate into identical improvements of LODs and LOQs. LODs and LOQs substantially depend on noise inherent in a PTR-MS signal. This noise can be described by a Poisson distribution: the 1*σ* error in a measurement that is derived from counting a total of *N* ions is $\sqrt{N\bullet {\tau}^{-1}\ }$, with *τ* being the integration time [19, 25, 26]. Taking this into account, LODs and LOQs could effectively be improved by a factor of 2–4 when the instrument was switched from DC-mode to RF-mode. Hence, just determining increases in ion yields may lead to overestimation of the instrument performances for quantitative analysis. For real-life applications, e.g. trace gas analysis in breath, LODs and LOQs have to be determined to take into account all effects of DC field and RF voltage applied within the IF.

Especially in diseased states, breath VOC concentrations may change quickly and abruptly. Hence, only real-time monitoring can provide complete and comprehensive information from breath VOC analysis [27–29]. PTR-ToF-MS with integration time of ≥ 200 ms enables breath-resolved continuous monitoring of breath volatiles. In this pilot study, the range of detectable volatile substances was significantly enlarged through application of IF technology.

## Conclusion

The spectrum of detectable VOCs in real-time breath analysis was considerably enhanced through the application of IF technology. The IF can be tuned in order either to obtain the best operating conditions for a specific compound of interest or to realize operating conditions which represent the best compromise for the acquisition of a large number of compounds. In contrast to previous setups, the IF used in this study did not have major effects onto ion chemistry within the DT itself and therefore offers optimal conditions for VOC screening in biomedical applications.

## Electronic supplementary material

ESM 1 (PDF 926 KB)
