# Supplementary material for: Effects of modular ion-funnel technology onto analysis of breath VOCs by means of real-time mass spectrometry
Source: Anal Bioanal Chem. 2020 Aug 13;412(26):7131–40. doi: 10.1007/s00216-020-02846-8 (PMC7497501; doi:10.1007/s00216-020-02846-8)
Supplement: Supplementary file 1 — (PDF 926 KB) [file 216_2020_2846_MOESM1_ESM.pdf]

**Analytical and Bioanalytical Chemistry**

**Electronic Supplementary Material**

# Effects of modular ion-funnel technology onto analysis of breath VOCs by means of real-time mass spectrometry

Giovanni Pugliese, Felix Piel, Phillip Trefz, Philipp Sulzer, Jochen K. Schubert, Wolfram Miekisch

**Table S1** Details and operating conditions for the modular IF

| | |
|---|---|
| Number of electrodes | 12 (6 RF+; 6 RF-) |
| Length | 2.2 cm |
| I.D. reduction | 1 cm to 0.2 cm |
| Electrodes distance | 0.1 cm |
| Pressure | 2.3 mbar |
| Temperature | 75 °C |
| RF voltage | 40-200 $V_{p\text{-}p}$ |
| RF frequency | 4.5 MHz |
| DC field | 4.5-27 V/cm |

**Table S2** Experimental design conducted for the ion funnel characterization

| | $E_{drift}$ [V/cm] | RF voltage [$V_{p-p}$] | DC field [V/cm] |
|---|---|---|---|
| Dry samples | 66 | 40 | 4.5/ 9 / 13.5 / 18 / 22.5 / 27 |
| | | 80 | 4.5/ 9 / 13.5 / 18 / 22.5 / 27 |
| | | 120 | 4.5/ 9 / 13.5 / 18 / 22.5 / 27 |
| | | 160 | 4.5/ 9 / 13.5 / 18 / 22.5 / 27 |
| | | 200 | 4.5/ 9 / 13.5 / 18 / 22.5 / 27 |
| | 48 | 40 | 4.5/ 9 / 13.5 / 18 / 22.5 / 27 |
| | | 80 | 4.5/ 9 / 13.5 / 18 / 22.5 / 27 |
| | | 120 | 4.5/ 9 / 13.5 / 18 / 22.5 / 27 |
| | | 160 | 4.5/ 9 / 13.5 / 18 / 22.5 / 27 |
| | | 200 | 4.5/ 9 / 13.5 / 18 / 22.5 / 27 |
| Humid samples | 66 | 40 | 4.5/ 9 / 13.5 / 18 / 22.5 / 27 |
| | | 80 | 4.5/ 9 / 13.5 / 18 / 22.5 / 27 |
| | | 120 | 4.5/ 9 / 13.5 / 18 / 22.5 / 27 |
| | | 160 | 4.5/ 9 / 13.5 / 18 / 22.5 / 27 |
| | | 200 | 4.5/ 9 / 13.5 / 18 / 22.5 / 27 |
| | 48 | 40 | 4.5/ 9 / 13.5 / 18 / 22.5 / 27 |
| | | 80 | 4.5/ 9 / 13.5 / 18 / 22.5 / 27 |
| | | 120 | 4.5/ 9 / 13.5 / 18 / 22.5 / 27 |
| | | 160 | 4.5/ 9 / 13.5 / 18 / 22.5 / 27 |
| | | 200 | 4.5/ 9 / 13.5 / 18 / 22.5 / 27 |

**Table S3** Participant list with demographic parameters

| Volunteer ID | Sex | Age | Weight (Kg) | Height (cm) | Smoker |
|---|---|---|---|---|---|
| 01 | M | 30 | 78 | 175 | YES |
| 02 | M | 28 | 60 | 176 | NO |
| 03 | F | 38 | 80 | 165 | NO |
| 04 | M | 41 | 80 | 185 | NO |
| 05 | M | 37 | 85 | 187 | YES |
| 06 | M | 31 | 62 | 170 | YES |
| 07 | M | 39 | 85 | 192 | NO |
| 08 | M | 35 | 105 | 195 | NO |
| 09 | M | 34 | 105 | 183 | NO |
| 10 | M | 28 | 68 | 180 | YES |
| 11 | F | 20 | 65 | 171 | YES |
| 12 | F | 32 | 58 | 175 | YES |
| 13 | M | 26 | 65 | 170 | YES |
| 14 | M | 30 | 75 | 180 | NO |
| 15 | M | 44 | 82 | 177 | NO |
| 16 | M | 19 | 95 | 171 | YES |
| 17 | M | 47 | 92 | 188 | NO |
| 18 | M | 40 | 64 | 170 | YES |
| 19 | M | 29 | 75 | 180 | NO |
| 20 | M | 48 | 105 | 195 | NO |
| 21 | M | 54 | 85 | 179 | YES |

a)

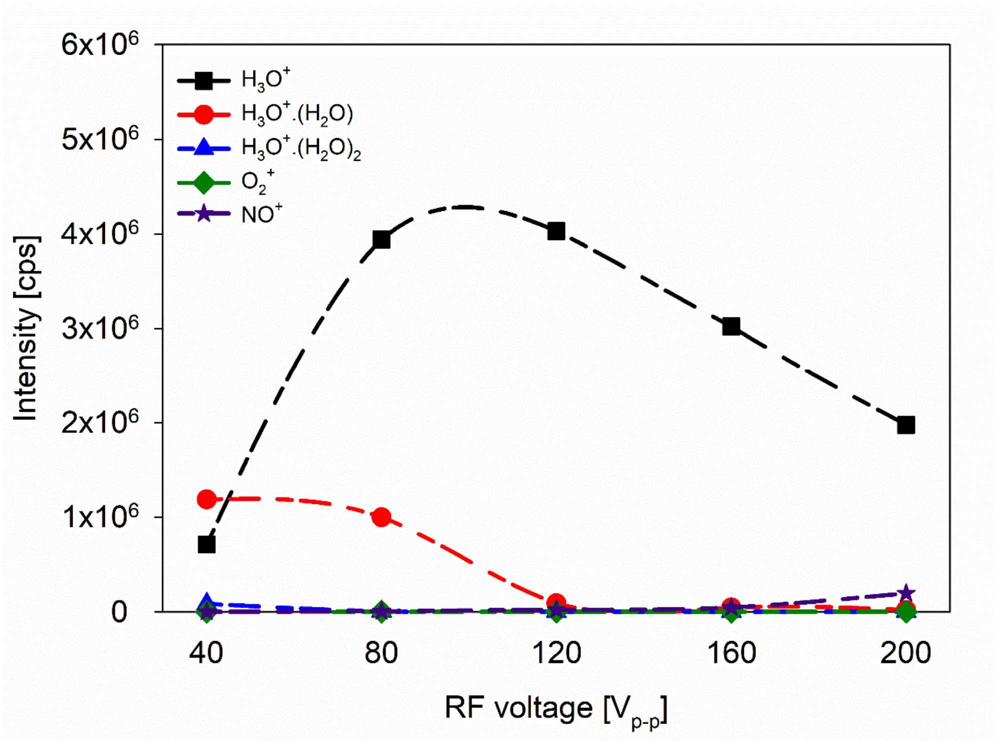


b)

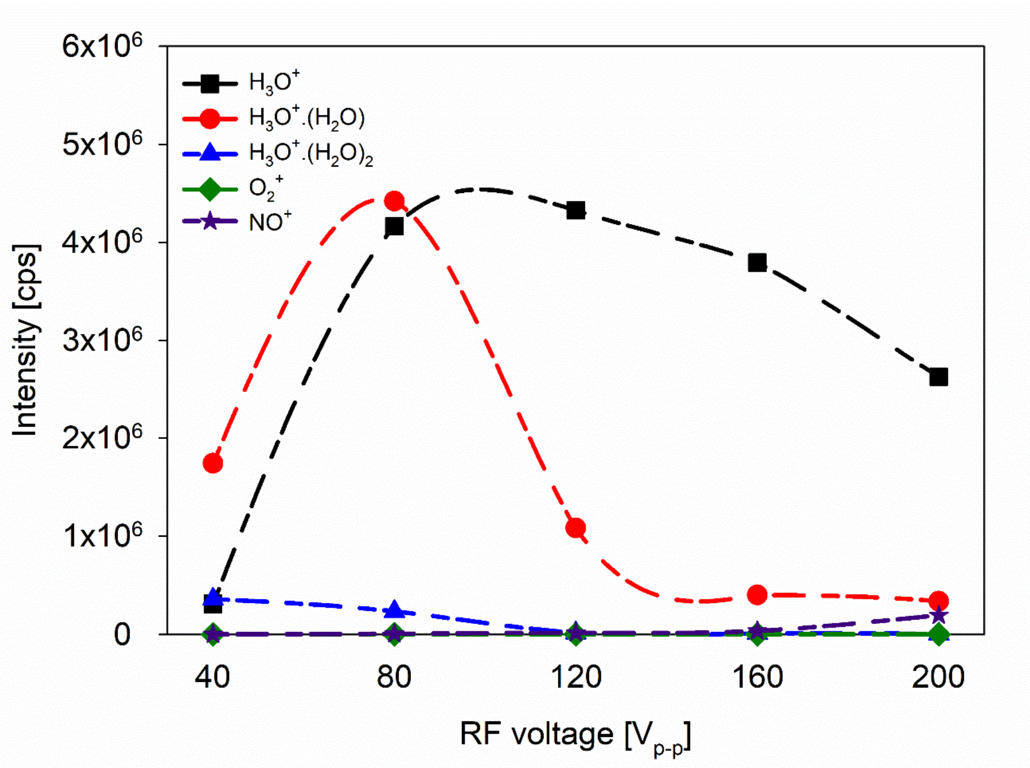


**Fig. S1** Ion intensities in counts per second (cps) of the water reagent ions ($H_3O^+.(H_2O)_n$, n = 0, 1 and 2) and as well as parasitic ions $O_2^+$ and $NO^+$ present in the drift tube under dry (a) and humid (b) conditions as a function of RF voltage. DC was 13.5 V/cm and $E_{drift}$ was 48 V/cm

a)

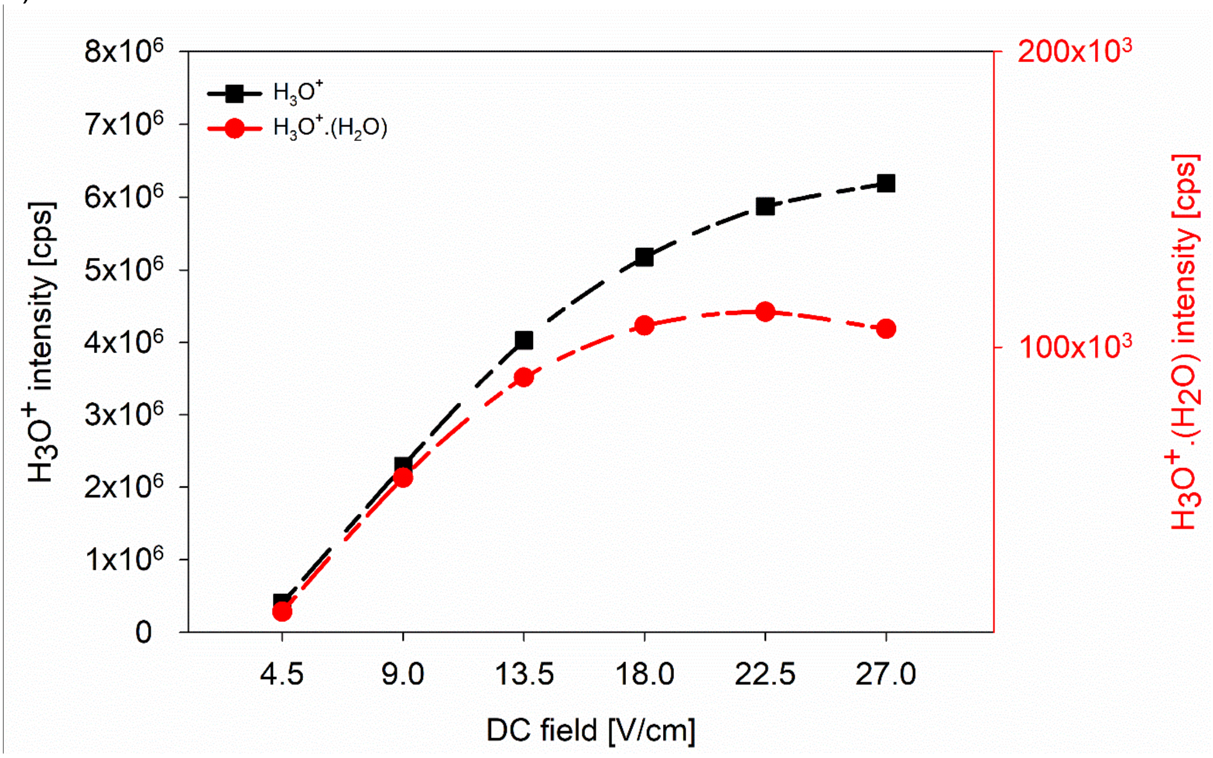


b)

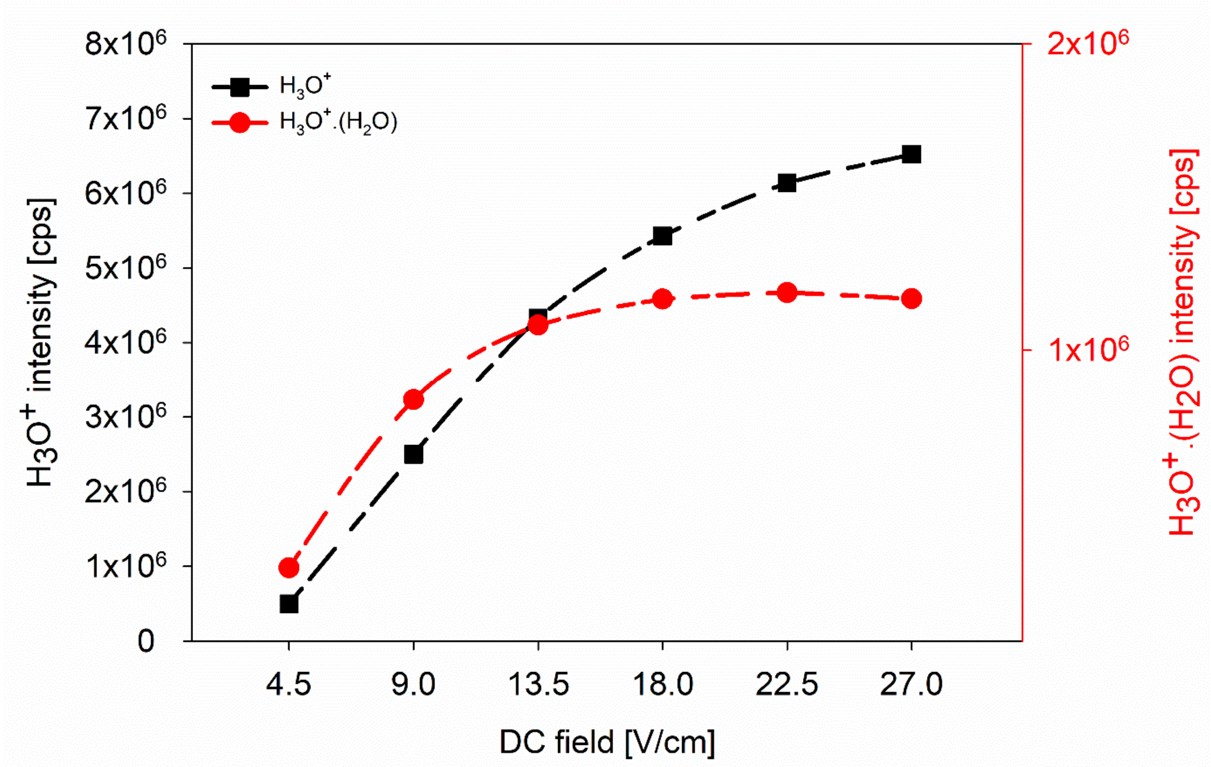


**Fig. S2** Ion intensities in counts per second (cps) of $H_3O^+$ and $H_3O^+.(H_2O)$ present in the drift tube under dry (a) and humid (b) conditions as a function of the DC field (V/cm). RF voltage was 120 $V_{p-p}$ and $E_{drift}$ was 48 V/cm

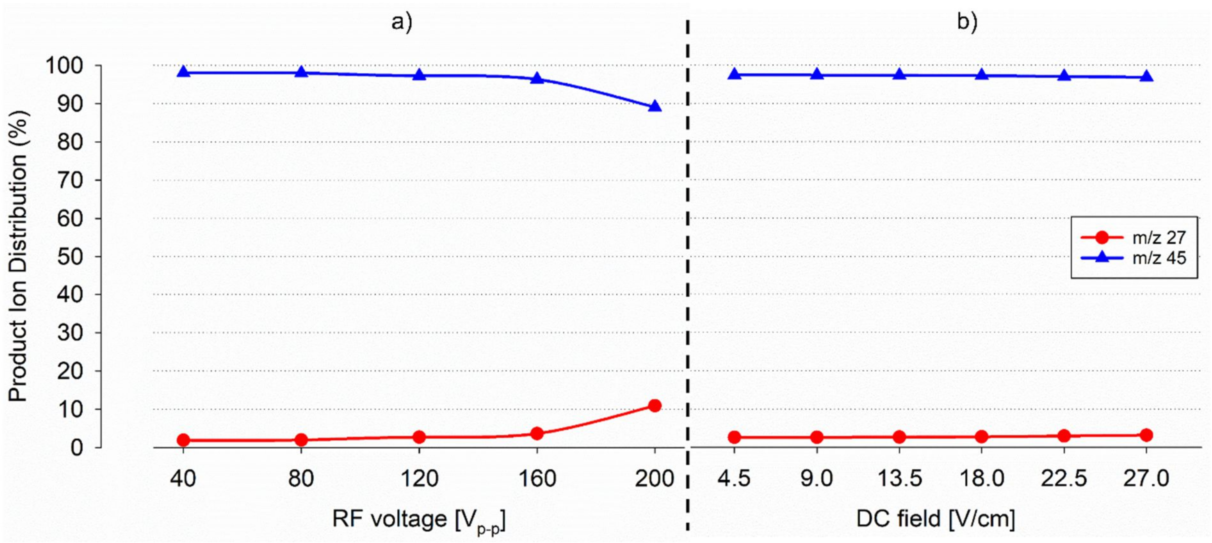


**Fig. S3** Relative signal variation for protonated acetaldehyde ($C_2H_5O^+$, *m/z* 45) and its fragment ($C_2H_3^+$, *m/z* 27) as function of RF voltage (panel a; DC = 13.5 V/cm, $E_{drift}$ = 66 V/cm) and DC voltage (panel b; RF = 120 $V_{p\text{-}p}$, $E_{drift}$ = 66 V/cm)
